# RADIOGRAPHIC UNION OVERESTIMATES BIOMECHANICAL STRENGTH: A CEILING EFFECT OF THE RUST SCORE IN RAT TIBIA MODELS

**DOI:** 10.1590/1413-785220263406e307811

**Published:** 2026-11-09

**Authors:** Ana Letícia Pagotti Rubello, Ricardo Martins-Santos, Iverson Silva Correia, Nilza Alzira Batista, José Ricardo Lenzi Mariolani, André Luís Lugnani de Andrade, William Dias Belangero

**Affiliations:** 1 Universidade Estadual de Campinas (UNICAMP), Laboratorio de Biomateriais em Ortopedia (LABIMO), Campinas, SP, Brazil.; 2 Universidade Estadual de Campinas (UNICAMP), Faculdade de Ciencias Medicas, Hospital de Clinicas da Unicamp, Campinas, SP, Brazil.

**Keywords:** Tibial Fractures, Fracture Healing, Radiography, Biomechanical Phenomena, Rats, Wistar, Fraturas da Tíbia, Consolidação da Fratura, Radiografia, Fenômenos Biomecânicos, Ratos Wistar

## Abstract

**Objective:**

To evaluate if the Radiographic Union Scale for Tibial Fractures (RUST) identifies bone consolidation in rat tibia osteotomies, correlating it with biomechanical parameters.

**Methods:**

Forty-five Wistar rats underwent right proximal tibia osteotomies, fixed with 0.8mm Kirschner wires. At 8 weeks, tibias were radiographed and underwent three-point bending tests to determine maximum force recovery (Frec). Biomechanical union was Frec > 80% of the intact side. Five blinded orthopedists scored radiographs using RUST (4-12); scores ≥ 10 indicated radiographic union.

**Results:**

Thirty-eight rats were analyzed. Spearman's correlation for non-normal data showed no significant relationship between absolute RUST scores and Frec (ρ = 0.09; p = 0.58). Fisher's Exact test revealed no association between RUST ≥ 10 and Frec > 80% (p = 0.50). While 36 tibias (94.7%) achieved RUST ≥ 10, only 16 (42.1%) reached the biomechanical threshold.

**Conclusion:**

At 8 weeks, RUST ≥ 10 overestimates structural recovery and cannot reliably predict >80% force restoration in this model. Radiographic bridging precedes complete biomechanical maturation, indicating a scoring system ceiling effect. **Level of Evidence V; Animal Experimental Study**.

## RESUMO

**Objetivo:**

Avaliar se a escala RUST (Radiographic Union Scale for Tibial Fractures) identifica a consolidação óssea em osteotomias de tíbia de ratos, correlacionando-a com parâmetros biomecânicos.

**Métodos:**

Quarenta e cinco ratos Wistar sofreram osteotomia proximal da tíbia direita, fixada com fio de Kirschner de 0,8 mm. Às oito semanas, as tíbias foram radiografadas e submetidas a ensaio de flexão em três pontos para medir a recuperação da força máxima (Frec). Consolidação biomecânica foi Frec > 80% do lado íntegro. Cinco ortopedistas avaliaram cegamente as imagens (RUST 4-12); escore ≥ 10 definiu consolidação radiográfica.

**Resultados:**

Analisou-se 38 animais. A correlação de Spearman (dados não-normais) indicou ausência de relação significativa entre os escores absolutos de RUST e Frec (ρ = 0,09; p = 0,58). O Teste Exato de Fisher não evidenciou associação entre RUST ≥ 10 e Frec > 80% (p = 0,50). Embora 36 tíbias (94,7%) tenham atingido o critério radiográfico (RUST ≥ 10), apenas 16 (42,1%) alcançaram o limiar biomecânico.

**Conclusão:**

Na oitava semana, o escore RUST ≥ 10 superestima a recuperação estrutural e não prediz de forma confiável uma recuperação superior a 80% da força neste modelo. A ponte óssea radiográfica precede a maturação biomecânica, evidenciando um efeito teto (ceiling effect) da escala. **Nível de Evidência V; Estudo Experimental em Animais**.

## INTRODUCTION

The assessment of bone healing is a cornerstone of both clinical practice and orthopedic research. Historically, the evaluation of fracture union relied on subjective radiographic interpretations, leading to low inter-observer reliability. To address this, the Radiographic Union Scale for Tibial Fractures (RUST) was developed as a standardized tool to quantify healing based on the presence of bridging callus and the visibility of the fracture line across four cortices in two orthogonal views.^1^ The score ranges from 4 (no healing) to 12 (complete consolidation), providing a more objective measure than general clinical impression.^1^

In clinical settings, the RUST score has proven to be a robust prognostic marker. Mundi et al. demonstrated that RUST scores obtained in three months post-operatively are significantly associated with the risk of nonunion in tibial shaft fractures, where lower scores accurately predict the need for secondary interventions.^2^ However, the translation of this scale to pre-clinical research is essential, as animal models remain the gold standard for testing new osteogenic therapies and fixation devices.^3^ Tawonsawatruk et al. validated the RUST scale in rat tibial fractures, reporting excellent inter-observer and intra-observer agreement, which supports its use in small animal models.^3^

Despite its reliability in identifying bridging callus, the correlation between radiographic appearance and the actual physical strength of the bone remains a subject of investigation. While some studies suggest that RUST correlates with structural properties observed in micro-computed tomography (micro-CT), others point to a complex relationship with biomechanical data.^4,5^ Alentado et al. found that radiographic scores in murine models correlate with micro-CT metrics such as bone volume fraction but emphasized that imaging alone may not fully capture the mechanical integrity of the healing callus.^6^ Furthermore, Cooke et al. highlighted that although RUST scores generally increase with bone stiffness and strength, there is significant variability in biomechanical properties within the same RUST score group.^5^

Defining a clear “union” threshold is critical for experimental consistency. Litrenta et al., using a sheep model, suggested that a RUST score of 10 or higher represents a reliable threshold for biomechanical union.^7^ However, assessing if this threshold translates universally to smaller rodents is necessary. Therefore, the present study aimed to evaluate the correlation between RUST scores and maximum force values in a rat tibia osteotomy model, specifically testing whether a categorical threshold can accurately predict biomechanically consolidated bones at 8 weeks.

## MATERIALS AND METHODS

The study protocol was approved by the Institutional Animal Care and Use Committee (CEUA-UNICAMP) under protocol numbers 6327-1/2023 and 6515-1/2024. Forty-five male Wistar rats were used in this experimental study. Each animal underwent a proximal metadiaphyseal osteotomy of the right tibia, which was stabilized using a 0.8 mm Kirschner wire. The animals were followed for a period of 8 weeks to allow for natural healing process.

At the 8-week endpoint, the animals were euthanized, and the right (operated) and left (intact) tibias were harvested. Digital radiographs were obtained in anteroposterior and lateral views with a focus-film distance of 72 cm. The radiographic images were then randomly assigned to five blinded orthopedic trauma specialists for scoring according to the RUST criteria (score 4 to 12). Consolidation was radiographically defined as a RUST score ≥ 10.^7^

Immediately following imaging, the tibias underwent biomechanical testing using a three-point bending assay in a universal testing machine (Intermetric, model IM-100). This setup allowed for the measurement of structural properties at the osteotomy site (Figure 1). The maximum force (Fmax) supported by each bone was recorded. To standardize the results, the percentage of force recovery was calculated for each animal as Frec (Fmax operated / Fmax intact) × 100. Based on established experimental protocols, biomechanical consolidation was defined as the recovery of more than 80% of the maximum force of the intact contralateral limb.^4,6^

**Figure 1:**
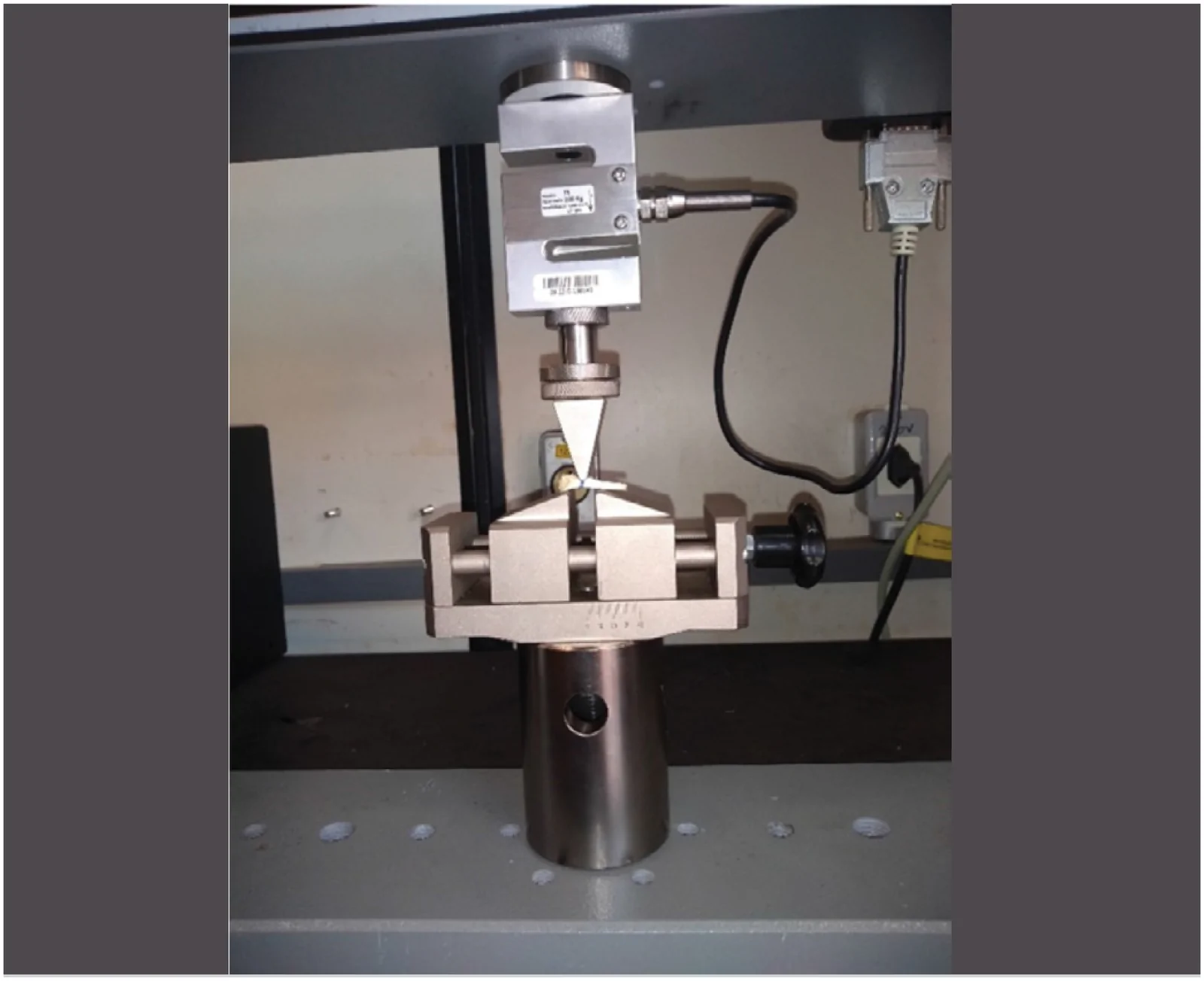
Experimental setup for the three-point bending test of the rat tibia.

Statistical analysis began with the Shapiro-Wilk test to assess data normality. Given the non-normal ordinal distribution of the radiographic scores, the non-parametric Spearman's rank correlation coefficient (ρ) was utilized to evaluate the relationship between absolute RUST scores and Frec. Additionally, Fisher's Exact test was employed to analyze the association between the categorical variables “radiographically consolidated” (RUST ≥ 10) and “biomechanically consolidated” (Frec > 80%), with significance set at p < 0.05.

## RESULTS

Thirty-eight animals were included in the final analysis after the exclusion of seven subjects due to infection or surgical complications. The biomechanical assays demonstrated a wide range of force recovery across the subjects at 8 weeks. The individual values for maximum force recovery (Frec) and absolute RUST scores for all evaluated subjects are detailed in Table 1.

**Table 1:** Maximum force results obtained in three-point bending tests for both operated and intact tibias in newton (N), maximum force recovery (Frec), and final RUST scores for each animal.

Rat ID	F_max operated_ (N)	F_max intact_ (N)	Frec (%)	RUST Score
1	145.3	182.8	79.5	9
2	208.6	182.7	114.2	10
3	124.7	169.4	73.6	12
4	166.3	153.8	108.1	12
5	167.6	218.1	76.8	12
7	66.7	108.7	61.4	11
8	142.7	181.4	78.7	10
9	136.6	154.7	88.3	10
11	184.0	198.8	92.6	10
12	84.9	181.7	46.7	10
13	44.6	186.0	24.0	10
14	99.1	148.2	66.9	12
15	137.7	168.1	81.9	11
16	132.3	200.6	66.0	11
17	172.1	244.1	70.5	10
19	100.5	195.8	51.3	9
20	179.4	196.1	91.5	11
21	219.1	193.4	113.3	12
25	82.9	212.4	39.0	12
26	153.9	183.3	84.0	12
27	82.2	245.3	33.5	12
28	103.6	143.9	72.0	11
30	94.6	190.3	49.7	10
31	120.3	148.8	80.8	10
32	134.6	139.1	96.8	11
33	150.7	176.5	85.4	12
34	203.5	199.9	101.8	11
35	120.4	185.5	64.9	10
36	100.6	167.2	60.2	11
37	115.2	123.8	93.1	12
38	110.0	169.5	64.9	12
39	111.0	127.9	86.8	11
40	112.4	184.1	61.1	12
41	128.6	147.8	87.0	12
42	116.5	153.5	75.9	12
43	100.0	173.3	57.7	12
44	144.4	167.4	86.3	12
45	19.3	183.9	10.5	11

Radiographic evaluation revealed that most samples presented significant bridging callus at 8 weeks. Representative images demonstrating the morphology of the tibias are shown in Figure 2.

**Figure 2:**
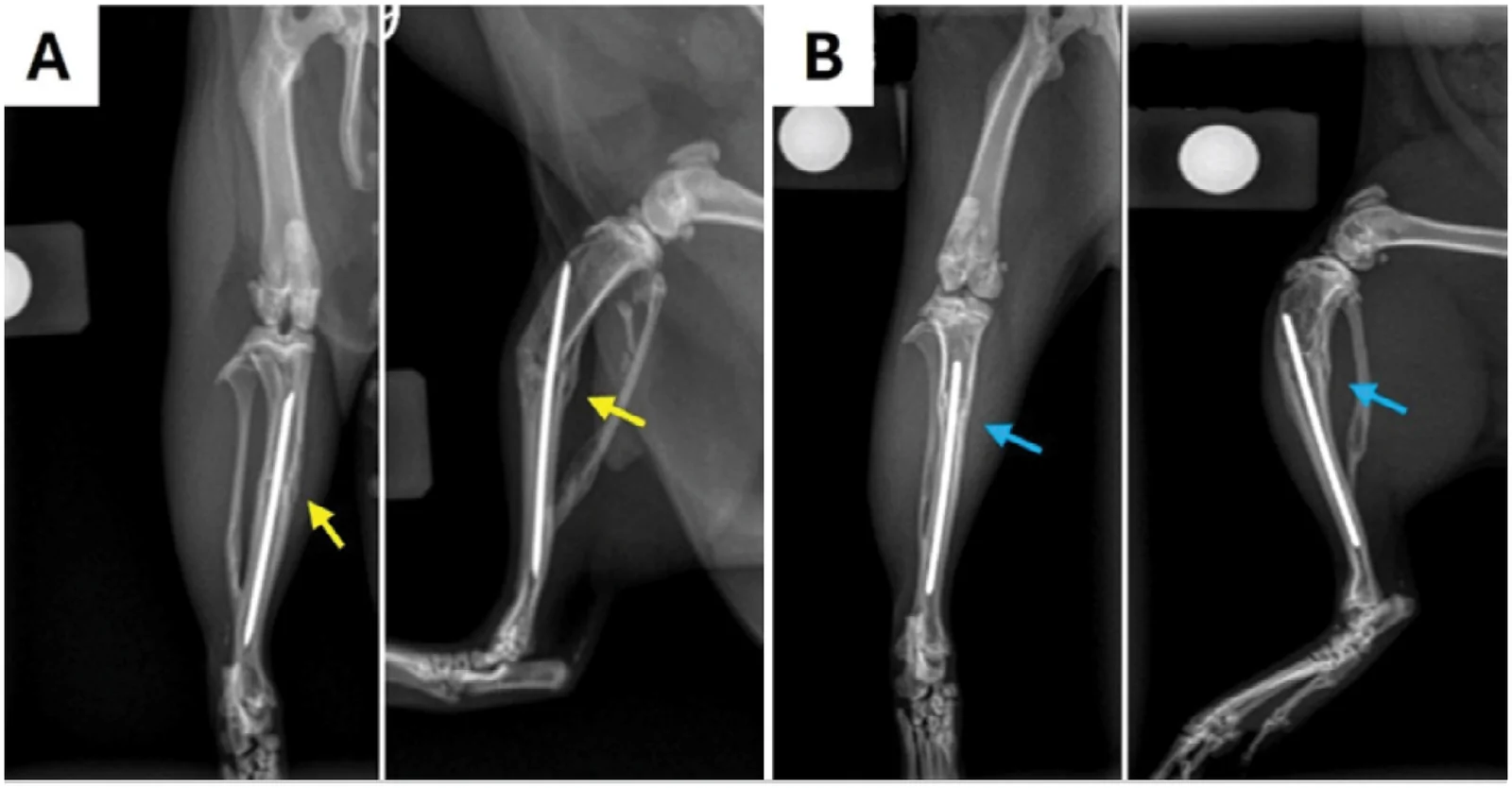
Representative radiographic images of rat tibias at 8 weeks post-osteotomy. (A) Sample with a RUST score < 10, showing persistent fracture lines (yellow arrows). (B) Consolidated sample with a RUST score ≥ 10, demonstrating robust bridging callus across multiple cortices (blue arrows).

The continuous data analysis revealed the absence of a significant correlation. The Spearman's rank correlation coefficient between the absolute RUST scores and maximum force recovery was ρ = 0.09 (p = 0.58). This dispersion is visually represented in the scatter plot in Figure 3.

**Figure 3:**
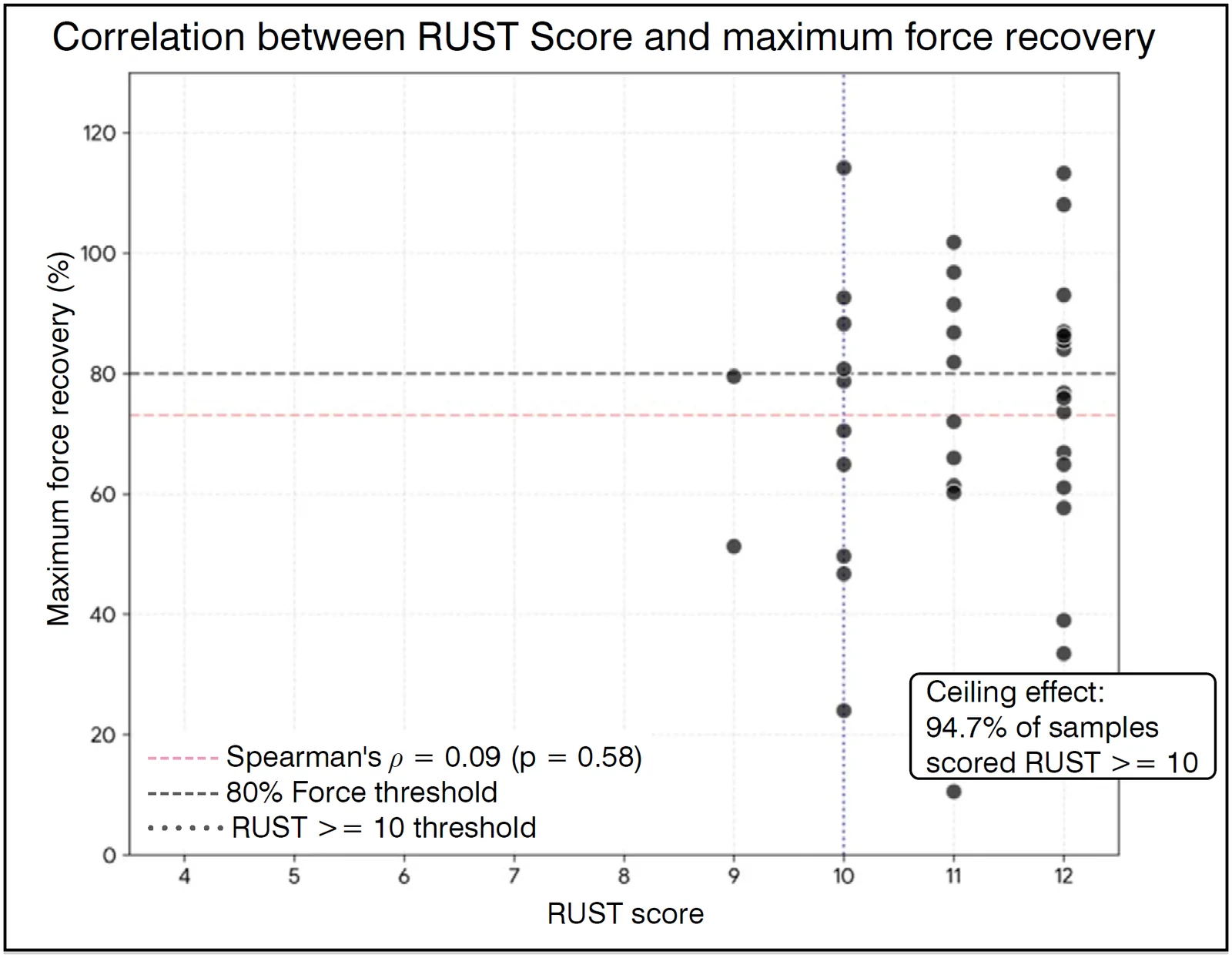
Correlation between RUST Score and Maximum Force Recovery.

* Black dots: represent individual data points for each evaluated rat tibia. Red dashed horizontal line: represents the mean force recovery reference line (Spearman's ρ = 0.09; p = 0.58). Gray dashed horizontal line: demarcates the 80% maximum force recovery (Frec) threshold. Blue dotted vertical line: demarcates the RUST ≥ 10 threshold.

The categorical analysis revealed a substantial discrepancy between radiographic and biomechanical findings. Out of the 38 rats, 36 (94.7%) achieved a RUST score of 10 or higher. However, only 16 rats (42.1%) achieved the biomechanical consolidation threshold (> 80% Frec). Fisher's Exact test revealed no significant association between achieving a RUST score ≥ 10 and recovering > 80% of Frec (p = 0.50). The overwhelming frequency of high RUST scores indicated a ceiling effect, preventing the radiographic system from effectively distinguishing between biomechanically robust and weak tibias at this time point.

## DISCUSSION

The primary finding of this study is that at 8 weeks post-osteotomy in a rat tibia model, a RUST score of 10 or higher overestimates actual structural recovery. While the scale reliably evaluates the visible presence of a bony bridge, it did not significantly predict whether the bone had recovered at least 80% of its original biomechanical strength (p = 0.50).

These findings illuminate a critical phenomenon in pre-clinical fracture models: radiographic union precedes biomechanical maturation. In our cohort, 94.7% of the animals exhibited sufficient cortical bridging to score a RUST of 10 or higher, yet less than half possessed the mechanical integrity to withstand 80% of the physiological load in bending. This biological discrepancy creates a “ceiling effect” for the RUST scale at 8 weeks, where numerical scores cluster at the top of the scale despite underlying structural weakness.

This lack of strong correlation (ρ = 0.09) is consistent with the observations made by Cooke et al. regarding toughness, who reported that biological variability within each RUST score is substantial.^5^ As they noted, mechanical properties such as ultimate strength are heavily influenced by the mineral density and mature ultra-structure of the callus, details that are not fully captured by two-dimensional plain radiographs.^5^ Two bones with an identical RUST score of 10 may look similar regarding the *presence* of bridging but vastly differ in the *quality* of that bridge.^4,5^

Our results provide an important counterpoint to large-animal studies. For example, Litrenta et al. found that a RUST score of 10 correlated well with a recovery of 90% of torsional stiffness in a sheep model, successfully defining union.^7^ However, the metabolic rate of rodents leads to a much faster initial bridging phase.^4^ Fiset et al. demonstrated through micro-CT and torsion testing in rats that while RUST accurately reflects the initial progression of healing, actual mechanical stability is highly dependent on the later stages of remodeling.^4^ Our data empirically validates this concept; seeing bone on an X-ray is not synonymous with functional load-bearing capacity in early-stage rodent models.

The validation of RUST in small animals remains high regarding inter-observer reliability, as shown by Tawonsawatruk et al.^3^ and Alentado et al.^6^ The scale is excellent for confirming that the healing cascade is successfully underway. However, researchers must be cautious. Since radiographic evaluation is nondestructive, it is tempting to use it as a standalone proxy for terminal biomechanical testing.^6^ Our results show that relying exclusively on a RUST ≥ 10 threshold to define “complete union” at 8 weeks in rats will result in false positives regarding structural strength. Researchers utilizing the rat tibia model should be aware of the temporal lag between radiographic appearance and mechanical properties. While RUST is an essential tool for tracking early callus formation, biomechanical testing remains indispensable for confirming true functional consolidation.

The main strengths of this study include a robust sample size subjected to rigorous biomechanical testing and the use of a blinded panel of orthopedic specialists for image evaluation, ensuring high clinical relevance. Conversely, the limitations include the evaluation restricted to a single 8-week endpoint – which precludes longitudinal tracking of the healing timeline – and the reliance on 2D plain radiographs rather than 3D micro-CT for volumetric callus assessment.

## CONCLUSION

In this experimental rat model, neither absolute RUST scores nor the categorical threshold of ≥ 10 successfully predicted the recovery of > 80% maximum force at 8 weeks. Radiographic bridging occurs faster than full biomechanical maturation, leading to a ceiling effect where plain radiographs overestimate structural strength. Consequently, a RUST score ≥ 10 should not be used as an isolated surrogate marker for functional load-bearing consolidation in this specific pre-clinical time point.

## Data Availability

The contents are already available.
